# Supplementary material for: Unusual lizard fossil from the Miocene of Nebraska and a minimum age for cnemidophorine teiids
Source: R Soc Open Sci. 2020 Aug 5;7(8):200317. doi: 10.1098/rsos.200317 (PMC7481707; doi:10.1098/rsos.200317)
Supplement: Comparative skeletal specimens [file rsos200317supp1.docx]

**Unusual lizard fossil from the Miocene of Nebraska and a minimum age for cnemidophorine teiids**

Royal Society Open Science

Simon Scarpetta

Department of Geological Sciences, Jackson School of Geosciences, The University of Texas at Austin, Austin, TX, USA

**ESM 1**

**Comparative specimens**

List of comparative specimens used to identify Miocene teiid lizard fossil.

**Teiidae**

*Ameiva ameiva* UF 57894, 57896, 63937, 69018

*Ameiva praesignis* UF 135491

*Ameivula* *ocellifera* CAS 49378 [1]

*Aurivela* *longicauda* CAS 18414 [2]

*Aspidoscelis deppii* CAS 68970; TNHC 96783, 96795

*Aspidoscelis exsanguis* TxVP M-8468

*Aspidoscelis flagellicauda* TxVP M-8470, M-15027

*Aspidoscelis gularis* TxVP M-15028

*Aspidoscelis hyperythrus* TxVP M-15029, M-15030

*Aspidoscelis inornata* TxVP M-8471

*Aspidoscelis marmoratus* TxVP M-15031, TxVP M-15032

*Aspidoscelis neomexicana* UF 57181

*Aspidoscelis sexlineatus* TxVP M-7161

*Aspidoscelis* sp. (Travis County, Texas) TxVP M-278

*Aspidoscelis sonorae* TxVP M-8473, M-8481, M-8562, M-8627

*Aspidoscelis tigris* TxVP M-8469, M-8477, M-8482, M-8629, M-8630, M-8631, M-15033, M-15034, M-15035; UCMP 138075

*Aspidoscelis uniparens* TxVP M-8479, M-8480, M-8565

*Callopistes maculatus* MVZ 175858; TxVP M-8461; UF 61305

*Callopistes flavipunctatus* MVZ 82409; UF 76336

*Cnemidophorus lemniscatus* MVZ 83452; UF 76231, 99421, 99530, 99950

*Dicrodon guttulatum* MVZ 77474; UF 48447

*Dicrodon heterolepis* MVZ 85399

*Dracaena guianensis* CAS 225062

*Holcosus festivus* MVZ 79608

*Holcosus quadrilineata* UF 37170

*Holcosus undulatus* CAS 68659l; UF 51244

*Kentropyx borckiana* UF 99285

*Kentropyx pelviceps* MVZ 174864

*Medopheos edracanthus* UF 99684

*Pholidoscelis chrysolaemus* UF 99352, 99646

*Pholidoscelis lineolatus* UF 99354

*Pholidoscelis plei* UF 22260

*Pholidoscelis taeniurus* UF 99572

*Salvator merianae* UF 158009, 175135

*Salvator rufescens* MVZ 92987; UF 135285

*Teius teyou* MVZ 92947

*Tupinambis nigropunctatus* TxVP M-8632, M-8633

*Tupinambis teguixin* UF 22154, 153696

**Gymnophthalmidae + Alopoglossidae**

*Bachia peruana* CAS 93227

*Echinosaura panamensis* CAS 99994

*Colobosaura modesta* USNM 341978 (viewed on Digimorph.org, available at <http://digimorph.org/specimens/Colobosaura_modesta/>)

*Gymnophthalmus speciosus* CAS 98968

*Pholidobolus* *montium* FMNH 197865 (viewed on Digimorph.org, available at <http://digimorph.org/specimens/Pholidobolus_montium/>)

*Potamites ecleopus* MVZ 163399

**Lacertidae**

*Meroles anchietae* CAS 126110

*Meroles knoxii* CAS 173945

*Meroles suborbitalis* CAS 125821

*Lacerta viridis* CAS uncatalogued; TxVP M-9022; YPM 12858 (viewed on Digimorph.org, available at <http://digimorph.org/specimens/Lacerta_viridis/>)

*Ophisops elegans* CAS 105902

*Phoenicolacerta* (*Lacerta*) *laevis* TxVP M-15036

*Podarcis muralis* CAS uncatalogued

*Psammodromus algirus* CAS 92431

*Takydromus ocellatus* FMNH 255513 (viewed on Digimorph.org, available at <http://digimorph.org/specimens/Takydromus_ocellatus/>)

*Takydromus* cf. *sexlineatus* TxVP M-15037

*Teira* (*Lacerta*) *dugesii* CAS 94069

*Timon lepidus* (*Lacerta ocellata*) CAS 225587, 225588; TxVP M-8499

References:

1. Morphosource.org. 2020a *Ameivula ocellifera* (media number M45303-82564). ark:/87602/ m4/M82564. Accessed June 1, 2020. See http:// www.morphosource.org/Detail/MediaDetail/ Show/media_id/45303.

2. Morphosource.org. 2020b *Aurivela longicauda* (media number M45295-82556). ark:/87602/ m4/M82556. Accessed June 1, 2020. See http:// www.morphosource.org/Detail/MediaDetail/ Show/media_id/45295.
